# Complications and hospital stay after endoscopic retrieval of drug baggies in body stuffers: an observational prospective study

**DOI:** 10.1038/s41598-021-84898-z

**Published:** 2021-03-08

**Authors:** Mahtab Shabani, Marzieh Kefayati, Hossein Hassanian-Moghaddam, Nasim Zamani, Rebecca McDonald

**Affiliations:** 1 Private Gastreoentrologist, Tehran, Iran; 2 Department of Internal Medicine, Shahid Beheshti University of Medical Sciences, Tehran, Iran; 3 Social Determinants of Health Research Center, Shahid Beheshti University of Medical Sciences, Tehran, Iran; 4 Department of Clinical Toxicology, Loghman Hakim Hospital, School of Medicine, Shahid Beheshti University of Medical Sciences, South Karegar Street, Tehran, Iran; 5 National Addiction Centre, Institute of Psychiatry, Psychology and Neuroscience, King’s College London, Addiction Sciences, London, UK

**Keywords:** Gastroenterology, Risk factors

## Abstract

Body stuffers routinely receive conservative treatment, i.e. administration of the laxative polyethylene glycol for the passage of ingested drug baggies and observation. Endoscopic baggie removal may offer a safe alternative that could result in shorter hospitalization. We aimed to compare complications, hospital stay, and final outcome in body stuffers assigned to endoscopy versus conservative treatment. This is an observational prospective study of body stuffers presenting to a clinical toxicology center in Tehran (Iran) in 2016–2019, irrespective of the drug ingested. Eligible patients had baggies in their upper gastrointestinal tract and presented without severe poisoning. Patients received either endoscopy or conservative treatment, and clinical outcomes were compared between the groups. A total of 69 patients were enrolled, with a median age of 29 years (range 18–64), among whom 1 was female (2%). Eighteen and 51 patients were endoscopically and conservatively managed, respectively. Drugs most commonly ingested were heroin in endoscopy patients (8/18 cases; 44%) and methamphetamine in the conservative group (28/51 cases; 55%). Endoscopy patients had a shorter hospital stay (median 1.5 vs. 2 days, *P* = 0.018). In the conservative group, one patient died, and the rate of complications was significantly higher, with more patients experiencing side effects (OR = 1.4, 95% CI = 1.2, 1.7) and requiring intubation (OR = 1.3, 95% CI = 1.1, 1.5). Endoscopic retrieval was associated with fewer complications and shorter hospitalization. Endoscopy may be a safe treatment for body stuffers without severe poisoning on presentation.

## Introduction

Body stuffing refers to the practice of street-level drug dealers and/or users who ingest drugs in an unplanned attempt to avoid police discovery of drug possession. In body stuffing, the drugs are typically loosely wrapped, e.g. in plastic or aluminium foil^1^. By contrast, body packing is a common method of international drug trafficking where smugglers intentionally ingest a large number of high-density, drug-filled packages to transport them across borders.

Despite the smaller number of packages, the risk of rupture is greater in body stuffers as a result of inadequate wrapping material and can lead to fatal outcome due to the release of large amounts of toxic stimulants or opioids^2–5^. There are algorithms that dictate the management of body packers, but such guidelines remain scarce or non-validated for body stuffers^5–8^.

The UK Royal College of Emergency Medicine (RCEM) 2014 guidelines outline that body stuffing patients “should be admitted and observed for [at least] 6–8 h post ingestion. [T]he use of activated charcoal 50 g [should be considered] to reduce absorption of the drug, [and] the patient [managed] according to […] the drug ingested”^9^.

It is generally recommended to observe these patients during routine conservative treatment to facilitate the passage of the baggies^10^. Treatment (e.g. naloxone for opioid poisoning) is indicated when the patient is showing signs and symptoms of drug poisoning^11,12^. Patients with obstruction or those with severe poisoning, especially if they have ingested stimulants (e.g. cocaine or amphetamine), may need surgical interventions^9,13–17^. However, surgery poses significant risks, such as missing small drug baggies during the procedure or their rupture during intestinal milking, which may cause more severe or even life-threatening complications^18^.

Endoscopy has been endorsed as a treatment in patients poisoned from the ingestion of iron, batteries, and sustained-release medications^19,20^. However, the latest clinical guidelines of the European Society of Gastrointestinal Endoscopy explicitly state that endoscopy is not recommended for the retrieval of ingested drug packets in body packing^20^.

Nonetheless, there is emerging evidence from Australia, Turkey, the US, and our center in Iran that upper gastrointestinal endoscopy has been used successfully in body stuffers who had ingested opioids and methamphetamine^18,21–23^. These case reports suggest that endoscopy may be used in body stuffers to prevent more invasive treatment methods including surgery.

Literature on the use of endoscopy in body stuffers is limited to such case reports, and, to date, no head-to-head comparison of conservative methods versus endoscopy has been conducted. To address this evidence gap, we aimed to compare results and complications of (1) endoscopic retrieval of ingested drug baggies with (2) the routine conservative management of administration of polyethylene glycol (PEG) in body stuffers referred to our center.

## Materials and methods

### Process

In an observational prospective study between 2016 and 2019, ***all*** patients referred to our center with history of drug ingestion were identified. Patients were categorized into three different groups; asymptomatic, mild poisoning (mild agitation or loss of consciousness), and severe poisoning (vital sign abnormalities, seizure, severe agitation, or severe loss of consciousness mandating intubation).

Study participation was open to body stuffers who met the following inclusion criteria: a) All baggies were still in their upper gastrointestinal (GI) tract (esophagus and stomach), and b) they were asymptomatic or had mild poisoning.

Accordingly, patients were excluded if a) their baggies that had passed the pylorus, b) they had severe poisoning, or c) they were body packers. To this end, an abdominopelvic computed tomography (CT) scan was performed in all cases to determine if the patients were body packers (multiple high-density drug packages in the whole GI tract) or stuffers (small number of low-density baggies in the upper GI tract). The information obtained by CT (number/volume of packages, method of packaging) supplemented the history given by the patients or their accompanying police officer (incl. reported aim of drug ingestion).

For each patient, a trained physician completed a purpose-developed questionnaire containing information on the patients’ demographic characteristics (age and gender), background diseases (seizure, psychiatric diseases), history of drug abuse/addiction (based on DSM IV criteria), type and quantity of the substance of abuse in the baggies, time elapsed between drug ingestion and presentation (before possible endoscopy), CT results, urine confirmatory laboratory tests for drugs of abuse (immunoassay), treatment performed (conservative by administration of PEG and observation until complete passage of the baggies versus endoscopic retrieval), complications of the treatments (rupture, obstruction, severe poisonings), hospital stay, and final outcome.

Naloxone was given in cases of opioid poisoning without complications (e.g. aspiration pneumonia). Patients were only intubated when their loss of consciousness was severe (Glasgow Coma Scale < 8) or pulmonary complications such as aspiration occurred.

The patients were assigned to either the conservative (PEG) or the intervention (endoscopy) group based on the on-call days of the gastroenterology consultants, as only one of the three gastroenterologists at our center accepted to perform endoscopy to retrieve the baggies. Patients who were referred during the on-call shifts of the endoscopy-performing gastroenterologist received verbal explanation of the procedure they would undergo, subject to the patient’s written informed consent. In accordance with Iranian law, if the patient was referred by police, consent had to be taken from the patient him/herself as well as from a family member (i.e., this double consent requirement is intended to protect the patient’s right not to be forced to unwanted medical treatment). The family member received verbal explanation of the procedure over the phone and was then asked to present to the hospital and provide written informed consent.

#### Endoscopy treatment group

Patients in the endoscopy group were put on nil per os (NPO) regimen immediately and underwent endoscopy (GIHF 180 Olympus, Tokyo, Japan [12.8-mm gastroscope, 4-mm wire mono-filament with 2.6 mm diameter and 200 cm length] and 70-mm basket (ENDO-FLEX, Germany) within the first 6 h after admission. Midazolam (1 to 2.5 mg intravenously, 2 to 3 min before initiation of the procedure) and propofol (100 to 200 µg/kg/min) were administered to sedate the patients during the procedure.

**Figure 1:**
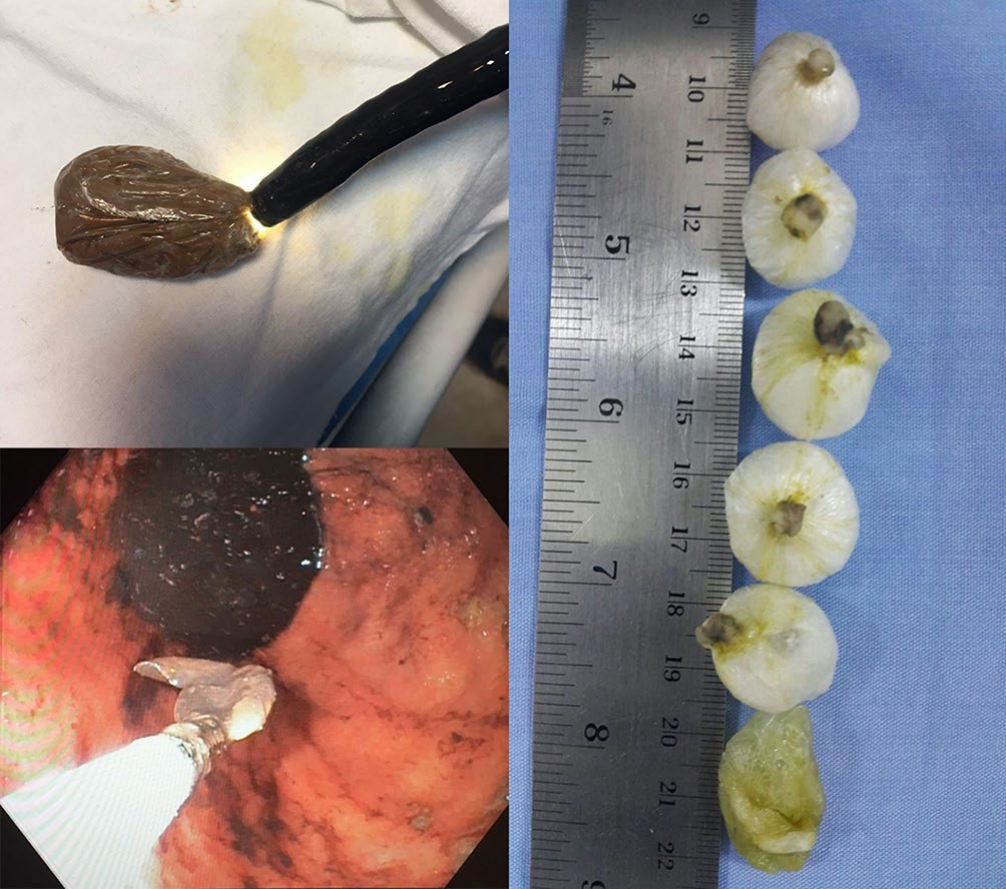
Lower left: Retrieved baggie by endoscope basket inside the stomach; upper left: Retrieved heroin baggie outside of the body after retieval; right: samples of methamphetamine baggies retrieved through endoscopy, the bottom baggie had opened in the stomach (prior to endoscopy).

The baggies were subsequently retrieved by the basket one by one (Fig. 1). The endoscopy procedure was performed in the operating room (OR) with the surgery team available in order to manage the patient as soon as possible if the baggies ruptured during the procedure.

#### Conservative treatment group

Patients in the conservative treatment group were treated by administration of PEG, antidote administration (if indicated), and surgery (if obstruction of the GI tract or baggie rupture occurred or the baggie remained in the same place in the GI tract for more than 72 h).

#### Patient observation and outcomes

Patients in both groups were monitored for potential complications (incl. GI obstruction, severe poisoning), their duration of hospital stay, and final outcome. In the endoscopy group, complications of anesthesia, aspiration, and perforation were also recorded.

### Statistical analysis

Statistical analysis was performed using SPSS Statistics for Windows, version 16.0 (SPSS Inc., Chicago, Ill., USA). The confounding factors were matched between the two groups and confirmed using non-parametric, Chi-Square and Fisher’s exact tests. Mann–Whitney U test was performed to compare the groups’ baggies, duration of hospital stay, time elapsed between ingestion and admission. Pearson Chi-Square was applied to compare severe complications including severe signs and symptoms of poisoning, GI obstruction, and need for surgery between the groups. A *P* value of less than 0.05 was considered to be statistically significant.

### Ethics approval and consent to participate

This study was supported by Social Determinants of Health Research Center, Shahid Beheshti University of Medical sciences, Tehran, Iran. All the experiment protocol for involving humans conducted in accordance with Declaration of Helsinki (1964), its revision (1975) and approved by Shahid Beheshti University of Medical Sciences ethics committee (IR.SBMU.RETECH.REC.1397.506, on 29-04-2016). Written informed consent statement obtained from all the participant to participate in the study.

### Consent to publish

Available.

## Results

A total of n = 69 patients were enrolled in the study and assigned to the endoscopy (n = 18) and conservative (n = 51) treatment groups, respectively.

**Figure 2:**
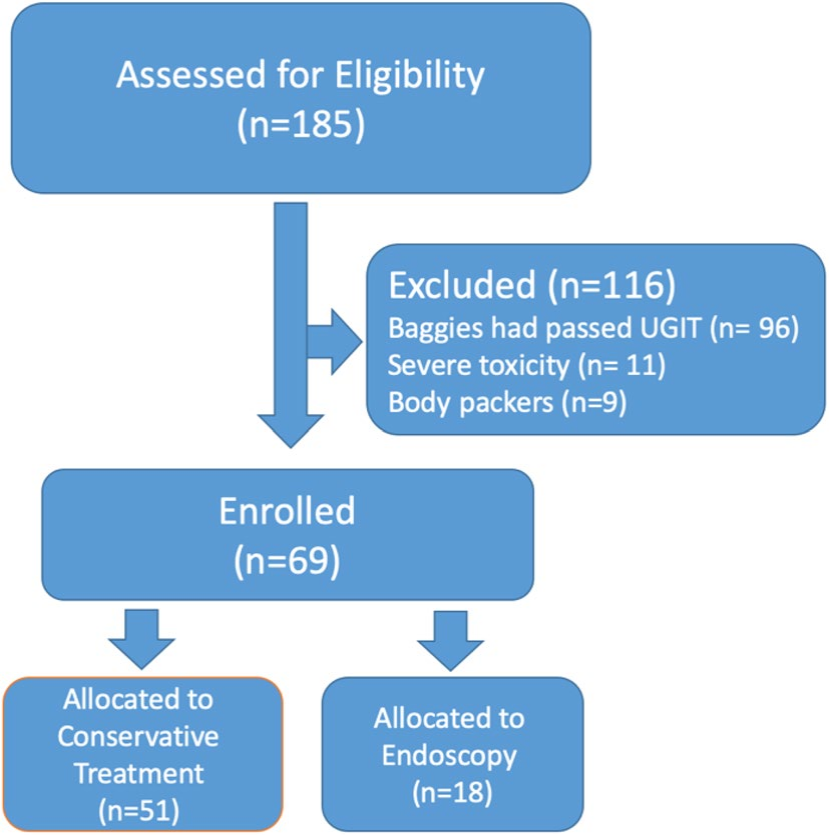
Algorithm of patient recruitment.

From the total of 185 cases who had ingested illicit drugs and were originally referred during the study period, 116 were excluded on the following grounds (see Fig. 2). In 96 cases, the drug bags had already passed pylorus (all or some of the baggies) by the time of the first abdominopelvic CT scan. Of the remaining 99 patients, 21 patients were excluded because they were suffering from severe poisoning. Another 9 patients were excluded because they were body packers. Stuffers in whom the CT confirmed the presence of baggies in the upper gastrointestinal tract (UGIT) remained in the study.

For the final study sample (n = 69; see Table 1), all potentially confounding factors including the patients’ age, gender, time of ingestion and number of the baggies, previous history of drug abuse/addiction, history of seizure and psychiatric disorders, medication history, symptoms at presentation, and their background diseases were similar between the two groups at presentation (i.e., p-value < 0.05).

**Table 1:** Demographic characteristics of body stuffers (n = 69) and presentation on arrival.

Variable		Endoscopy (n = 18)	Conservative (n = 51)	*p* value	Odds ratio (95% interval)
Median age (years) [IQR] (min, max)		30 [29, 35] (23, 56)	29 [25, 37] (18, 45)	0.193	–
Female gender n (%)		0	1 (2)	0.99 (F)	1.0 (0.1, 1.1)
Medication history n (%)		2 (11)	5 (10)	0.99 (F)	1.1 (0.2, 6.5)
Signs of poisoning n (%)	asymptomatic	10 (56)	29 (67)	0.92 (X^2^)	0.9 (0.3, 2.8)
	mild	8 (44)	22 (43)		
Median baggie number [IQR] (min, max)		1 [1, 3] (1, 12)	1 [2, 3] (1, 18)	0.526	–
Median time elapsed between ingestion and admission(h) [IQR] (min, max)		2 [1.4, 10] (0.5, 24)	2 [1, 3] (0.5, 24)	0.06	–
Median procedure time [IQR] (min, max)		16 [6, 26] (0.5, 65)	–	–	–
Drug content of baggies n (%)	Opium	2 (11)	3 (6)	0.60 (F)	2.0 (0.3, 13.1)
	Buprenorphine	1 (6)	2 (4)	0.99 (F)	1.4 (0.1, 16.9)
	Methamphetamine	6 (33)	28 (55)	0.12 (X^2^)	0.4 (0.1, 1.3)
	Hashish	2 (11)	1 (2)	0.16 (F)	6.2 (0.5, 73.6)
	Heroin	8 (44)	21 (41)	0.81 (X^2^)	1.1 (0.4, 3.4)
	Amphetamine	1 (6)	4 (8)	0.99 (F)	0.7 (0.1, 6.6)
	Methadone	0	1 (2)	0.99 (F)	1.0 (0.1, 1.1)
Drug dependency* n (%)	Opium	3 (17)	10 (22)	0.99 (F)	0.9 (0.2, 3.7)
	Methamphetamine	2 (11)	14 (27)	0.31 (F)	0.3 (0.1, 1.7)
	Amphetamine	3 (17)	2 (4)	0.09 (F)	5.4 (0.8, 35.9)
	Methadone	1 (6)	2 (4)	0.99 (F)	1.5 (0.1, 18.2)
	Heroin	3 (17)	15 (29)	0.52 (F)	0.5 (0.1, 2)
	Alcohol	1 (6)	1 (2)	0.44 (F)	3.1 (0.2, 53.6)
	None reported	3 (17)	3 (6)	0.18 (F)	0.3 (0.1, 1.7)
	No	6 (33)	13 (25)	0.341 (X^2^)	0.6 (0.2, 1.9)
On-arrival signs (%)	Tachycardia	1 (6)	5 (10)	0.99 (F)	0.5 (0.1, 5.0)
	Mydriasis	1 (6)	3 (6)	0.99 (F)	0.9 (0.1, 9.7)

F = Fisher’s exact test, X^2^ = Pearson Chi-Square.

*Poly substance abuse in some cases.

In the conservative group, the most common drug contained in the baggies was methamphetamine (28/51 cases; 55%) followed by heroin (n = 21; 41%), and amphetamine (n = 4; 8%). In patients who underwent endoscopy, the most common drug inside the baggies was heroin (8 cases; 44%) followed by methamphetamine (n = 6/18; 33%), opium and hashish (both n = 2; 11%) (see Table 1). Two of the patients in the conservative group had ingested more than one drug.

Median [IQR](range) time elapsed between ingestion of the baggies and admission was 2 [1.4, 10] (0.5–24) and 2 [1, 3] (0.5–24) hours in endoscopy and conservative groups (*P* = 0.06). The median number of baggies ingested was 1 [1, 3] (1–12) and 1 [2, 3] (1–18) in the two groups, respectively (*P* = 0.5).

The rate of total side effects was generally higher in the patients who had been conservatively managed (see Table 2, *P* = 0.01).

**Table 2:** Clinical outcomes of body stuffers (n = 69).

Variable		Endoscopy (n = 18)	Conservative (n = 51)	*p* value	Odds ratio (95% interval)
Complications during hospitalization n (%)	Gastrointestinal bleeding	0	1 (2)	0.99 (F)	1 (1, 1)
	Aspiration pneumonia	0	1 (2)	0.99 (F)	1 (1, 1)
	Seizure	0	2 (4)	0.99 (F)	1 (1, 1.1)
	Intubation	0	12 (23)	0.03 (F)	1.3 (1.1, 1.5)
	Loss of consciousness	0	10 (20)	0.05 (F)	1.2 (1.1, 1.4)
	Rhabdomyolysis	0	2 (4)	0.99 (F)	1 (1, 1.1)
	Tachycardia	0	6 (12)	0.33 (F)	1.1 (1, 1.3)
	Surgery	0	4 (8)	0.57 (F)	1.1 (1.0, 1.2)
	Side effects	0	15 (29)	0.01 (F)	1.4 (1.2, 1.7)
	Agitation	0	7 (14)	0.18	1.2 (1.0, 1.3)
Median admission length (day) [IQR] (min, max)		1.5 [1, 2] (0.5, 7)	2 [1, 4] (1, 11)	0.018	–
Death n (%)		0	1 (2)	0.99 (F)	1.0 (0.1, 1.1)

In terms of complications during hospitalization, the rates of gastrointestinal bleeding, aspiration pneumonia, seizure, and rhabdomyolysis did not significantly differ between the two groups, occurring in none of the endoscopy patients and at most 2 (4%) of the conservatively managed patients. Zero endoscopy patients and four patients (8%) in the conservative treatment group required surgery, but this group difference was not significant.

Among endoscopy patients, there was no report of baggie rupture, gastrointestinal perforation, or complications of anesthesia.

Need for intubation or loss of consciousness occurred in none of the endoscopy patients and, accordingly, was significantly less common (*P*s = 0.03 and 0.05, respectively).

Of the 51 patients who underwent conservative treatment, 12 (23%) were intubated, 10 (20%) experienced loss of consciousness, and seven (14%) showed agitation in the course of hospitalization due to rupture of their baggies.

Severe adverse events were less common among patients of the endoscopy group, who all survived. In the conservative treatment group, one patient died from methamphetamine poisoning.

The median hospitalization period was significantly shorter (*P* = 0.01) in the endoscopy group (1.5 days) compared to 2 days in the conservative treatment group (Table 2).

## Discussion

To the best of our knowledge, the current study is the first to compare endoscopy with the routine conservative management often recommended for body stuffers^24,25^.

Among the 18 endoscopy patients treated in this study, none experienced baggie rupture or required surgery. Moreover, there were no incidents of need for intubation or loss of consciousness among endoscopy patients, which was significantly lower than among the conservatively managed group. Notably, unlike previous studies, body stuffers in our study were eligible for endoscopy regardless of drug ingested, and endoscopy was successful across the full range of substances (i.e. opioids, stimulants, cannabis; see Table 1).

Our promising endoscopy results likely reflect both the experience of our gastroenterologist in managing body stuffers and the characteristics of the tools applied. Since the endoscopy basket was out of scope during the retrieval procedure, there was no correlation between the diameter of the scope and baggie retrieval. The spacious area between the basket arms prevented baggie rupture. The administration of the sedatives midazolam and propofol relaxed the muscles of the GI tract and further reduced the risk of baggie rupture.

All patients in the endoscopy group survived. In the conservatively managed group, one patient died from methamphetamine poisoning, which has been associated with a higher rate of severe outcomes than other stimulants^26^.

In 2016, Shabani and colleagues first reported the successful retrieval of opioid baggies using endoscopy in four body stuffers, and Cowan et al. had previously published a case report on endoscopical retrieval of a methamphetamine bag from a patient’s esophageal tract^18,21^.

Our findings extend and confirm our colleagues’ findings and challenge existing guidelines for the treatment of body stuffers.

In body stuffers, endoscopy is not a generally advocated method of treatment due to the following concerns: the patient is not fasting on admission, the endoscope passage may be difficult, and the baggies may rupture during use of the endoscope basket.

However, in body stuffers the size of the baggies is generally small enough to be easily caught and removed using the endoscope basket provided the gastroendoscopist is skilled enough to perform retrieval. Stuffers are often referred earlier (i.e. relative to the time point of ingestion) compared to body packers because in general they had no *intent* to ingest great quantities of high-concentrated drugs. This increases the odds of detection of the baggies in upper GI tract, which is a mandatory factor to perform endoscopy^27,28^.

Püschel and colleagues evaluated 224 body stuffers and concluded that induction of vomiting was the best method of baggie retrieval in body stuffers, as endoscopic intervention and laxatives might both cause complications^25^. However, more recent evidence has been shown that induction of vomiting can cause baggie rupture, and therefore can be as dangerous or even more hazardous than the administration of laxatives^29^. In 2011, a review by Albertson et al. recommended PEG administration in opioid body stuffers. They also limited the efficacy of charcoal to the first few hours after ingestion of the baggies^30^.

Haymann-Maier and colleagues evaluated 132 patients retrospectively and concluded that a nonsurgical approach seldomly accompanied poor outcomes in these patients although three of their patients finally needed surgery^31^. Shahnazi and colleagues evaluated body packers and stuffers but did not advocate endoscopic retrieval due to fear of rupture^32^.

In general, most experts try to avoid endoscopy due to the fear of baggie rupture although recent studies have suggested this technique as a potential treatment in body stuffers if the condition is optimum and the GI specialist performing the endoscopy is highly skilled^4^. Invasive surgical approaches are only recommended when the process of passage is delayed or complicated by rupture and endoscopy has not located the baggies^21^.

### Limitations of the study

We encountered several limitations while performing this study. Our sample size was limited by at least two factors: (1) delays in patient admission and diagnosis and (2) limited availability of the investigational treatment (endoscopy). Firstly, our center is a referral center and therefore, some patients were referred from elsewhere and admitted to our center with delay. Conversely, among patients admitted on time, some did not report their baggie swallowing on admission, but only disclosed this information at a later point, effectively delaying accurate diagnosis. Such delays meant that, by the time of CT scanning, the baggies had already passed the patients’ pylorus, and they were no longer eligible for study inclusion, thus limiting our sample size.

Secondly, endoscopy, i.e. the investigational treatment in this study, was only accessible to eligible patients during on-call shifts of the gastroenterologist/intensivist who conducted the procedure, which further limited the number of the patients undergoing the procedure. When approached about conducting endoscopy in body stuffers prior to start of the study, the other two gastroenterologists at our center denied due to concerns around possible baggie rupture.

Since all endoscopies in this study were performed by a single gastroenterologist, the generalizability of our findings to other settings is unclear.

Finally, the risk of complications and level of poisoning seen in our patients tends to be higher than in primary hospitals. This may be due to the fact that our center is a referral one which generally admits patients in poorer conditions or later in the course of their poisoning.

It is also possible that the content and packaging of baggies may vary across regions. This study was limited to Tehran and may not be applicable to other regions in which baggie rupture may be less hazardeous to the patient.

To reduce such potential for referral and selection bias, future research should compare endoscopy to conservative treatment in a multi-center, randomized trial design.

Our study establishes endoscopy as potential alternative method to conservative treatment that can be used to safely remove drug baggies from the UGIT.

## Conclusion

Preliminary evidence from our study suggests that endoscopic retrieval of the baggies trapped in the esophageal tract and stomach can be performed safely and effectively in body stuffers presenting without severe poisoning. This endoscopy method was associated with a shorter duration of hospital stay and improved patient outcomes, relative to conservative treatment.

A larger, randomized trial would be needed to confirm our results and assign causality.

## Data Availability

The data is all presented in the text.

## Abbreviations

CI: Confidence interval
CT: Computed tomography
DSM: Diagnostic and statistical manual of mental disorders
GI: Gastrointestinal
ICU: Intensive care unit
IQR: Inter quartile range
OR: Odds ratio
PEG: Polyethylene glycol
RCEM: The UK Royal College of Emergency Medicine
SPSS: Statistical package for social science
UGIT: Upper gastrointestibal tract

## References

[1] Jordan MT, Bryant SM, Aks SE, Wahl M (2009). A five-year review of the medical outcome of heroin body stuffers. J. Emerg. Med..

[2] Hassanian-Moghaddam H, Abolmasoumi Z (2007). Consequence of body packing of illicit drugs. Arch. Iran. Med..

[3] Zamani N, Hassanian-Moghaddam H (2018). Ingestion of lead-contaminated packs of opium. N. Engl. J. Med..

[4] Hassanian-Moghaddam H, Amraei F, Zamani N (2019). Management recommendations for body stuffers at emergency units. Arh Hig Rada Toksikol..

[5] Moreira M, Buchanan J, Heard K (2011). Validation of a 6-hour observation period for cocaine body stuffers. Am. J. Emerg. Med..

[6] Bronstein AC (2009). 2008 annual report of the American Association of Poison Control Centers' National Poison Data System (NPDS): 26th annual report. Clin. Toxicol. (Phila).

[7] Burns, M. J. Decontamination of poisoned adults. Available through: http://cursoenarm.net/UPTODATE/contents/mobipreview.htm?9/4/9281?source=see_link

[8] Yamamoto T, Malavasi E, Archer JR, Dargan PI, Wood DM (2016). Management of body stuffers presenting to the emergency department. Eur. J. Emerg. Med..

[9] Royal College of Emergency Medicine. Caring for adult patients suspected of having concealed illicit drugs. Available online at: https://www.rcem.ac.uk/docs/College%20Guidelines/5z1.%20Caring%20for%20adult%20patients%20suspected%20of%20having%20concealed%20illicit%20drugs%20(June%202014).pdf (2014).

[10] Ambe P, Weber SA, Schauer M (2012). Swallowed foreign bodies in adults. Dtsch Arztebl Int..

[11] Ikenberry SO, Kue TL, Andersen MA (2011). Management of ingested foreign bodies and food impactions. Gastrointest. Endosc..

[12] Dray X, Cattan P (2013). Foreign bodies and caustic lesions. Best Pract. Res. Clin. Gastroenterol..

[13] Sugawa C, Ono J, Taleb M (2014). Endoscopic management of foreign bodies in the upper gastrointestinal tract: a review. World J. Gastrointest. Endosc..

[14] Aks SE, Vander Hoek TL, Hryhorczuk DO, Negrusz A, Tebbett I (1992). Cocaine liberation from body packets in an in vitro model. Ann. Emerg. Med..

[15] Telford JJ (2005). Management of ingested foreign bodies. Can. J. Gastroenterol..

[16] Ríos, G., Rodríguez, L., Lucero, Y., Miquel, I., Arancibia, M. E., & Alliende, F. Endoscopic findings associated with button battery ingestion in children: do we need to change the protocol for managing gastric location? *Pediatr. Emerg. Care* **36**(11), 523–526 (2018). 10.1097/PEC.0000000000001415 29369264

[17] Atiq M, Dang S, Olden KW, Aduli F (2008). Early endoscopic gastric lavage for acuteiron overdose: a novel approach to accidental pill ingestions. Acta Gastroenterol. Belg..

[18] Shabani M, Zamani N, Hassanian-Moghaddam H (2016). Endoscopic retrieval of baggies in body stuffers. Endosc. Int. Open.

[19] Höjer J, Personne M (2008). Endoscopic removal of slow release clomipramine bezoars in two cases of acute poisoning. Clin. Toxicol. (Phila).

[20] Birk M, Bauerfeind P, Deprez PH (2016). Removal of foreign bodies in the upper gastrointestinal tract in adults: European Society of Gastrointestinal Endoscopy (ESGE) Clinical Guideline. Endoscopy.

[21] Cowan T, Gibson R, Berling I (2015). Endoscopic treatment of upper gastrointestinal obstruction after ingestion of illicit drug packets. Clin. Gastroenterol. Hepatol..

[22] Asıl M, Dertli R (2017). Successful endoscopic treatment of an unusual foreign body in the stomach: a package of heroin. Ulus Travma Acil Cerrahi Derg..

[23] Durrani, M., Dugas, C., & Dasgupta, S. A curious case of the persistent body stuffer. *Case Rep. Emerg. Med*. Article ID 3948054 (2019). 10.1155/2019/3948054 PMC6766140 31637063

[24] Bahrami-Motlagh H, Hassanian-Moghaddam H, Behnam B, Arab-Ahmadi M (2015). Failure of surgical treatment in methamphetamine body-stuffers. J. Forensic. Leg. Med..

[25] Püschel K, Bachmann D (2007). Proving possession of drugs in so-called body stuffers. J. Forensic. Leg. Med..

[26] West PL, McKeown NJ, Hendrickson RG (2010). Methamphetamine body stuffers: an observational case series. Ann. Emerg. Med..

[27] Bahrami-Motlagh H, Mahboubi-Fooladi Z, Salevatipour B, Hassanian-Moghaddam H, Mirhashemi SH (2017). Comparison of low dose and standard dose abdominal CT scan in body stuffers. Clin. Toxicol. (Phila).

[28] Pollack CV, Biggers DW, Carlton FB (1992). Two crack cocaine body stuffers. Ann. Emerg. Med..

[29] de Bakker JK, Nanayakkara PW, Geeraedts LM, de Lange ES, Mackintosh MO, Bonjer HJ (2012). Body packers: a plea for conservative treatment. Langenbecks Arch. Surg..

[30] Albertson TE, Owen KP, Sutter ME, Chan AL (2011). Gastrointestinal decontamination in the acutely poisoned patient. Int. J. Emerg. Med..

[31] Heymann-Maier L, Trueb L, Schmidt S (2017). Emergency department management of body packers and body stuffers. Swiss. Med. Wkly..

[32] Shahnazi M, Hassanian-Moghaddam H, Gachkar L (2015). Comparison of abdominal computed tomography with and without oral contrast in diagnosis of body packers and body stuffers. Clin. Toxicol. (Phila).

